# Efficacy of Modified Upadacitinib Dosing in Pediatric Refractory IBD: A Case-Based Analysis

**DOI:** 10.3390/children12091268

**Published:** 2025-09-21

**Authors:** Frank Risto Rommel, Christa Bergheim, Inga Jerrentrup, Stefanie Weber, Andreas Jenke

**Affiliations:** 1 Department of General Pediatrics, University Children’s Hospital Marburg, University of Marburg, 35408 Marburg, Germany; frank.rommel@uk-gm.de (F.R.R.); stefanie.weber@med.uni-marburg.de (S.W.); 2 Department of Neonatology and Pediatric Gastroenterology, Children’s Hospital Kassel, Klinikum Kassel, 34125 Kassel, Germany; 3 Department of Medicine, Faculty of Health, University of Witten/Herdecke, 58455 Witten, Germany

**Keywords:** VEO-IBD, IBD, children, upadacitinib, JAK inhibitor, rescue therapy

## Abstract

This case report describes four pediatric patients with severe, therapy-refractory ulcerative colitis (UC) and one pediatric patient with severe, therapy-refractory Crohn’s disease (CD), all of whom failed widely used biologic therapies, including the approved treatments infliximab and adalimumab, as well as off-label use of vedolizumab and ustekinumab. Despite these first- and second-line interventions, patients’ diseases remained active. Following the initiation of upadacitinib, an off-label Janus kinase (JAK) inhibitor with positive results in adult UC and CD patients, four children experienced rapid symptom improvement and achieved clinical remission. The rising incidence of pediatric inflammatory bowel disease (IBD) and the limited number of approved therapies underscore the need for additional treatment options in pediatric gastroenterology. Infliximab remains one of the only biologic therapies approved for pediatric UC and CD, forcing clinicians to rely on off-label medications, such as vedolizumab, ustekinumab, and upadacitinib, when standard treatments fail. To address this gap, it is crucial to include pediatric patients in clinical trials of new therapies, expanding the range of approved medications and improving outcomes for children with IBD.

## 1. Introduction

Ulcerative colitis (UC) and Crohn’s disease (CD) are chronic inflammatory bowel diseases (IBD) that present significant therapeutic challenges, particularly in pediatric patients with severe, refractory forms [1]. Managing pediatric IBD is complex due to the limited availability of approved treatments and the unique physiological considerations of younger patients. The rising incidence of UC and CD in individuals under 18, including very young children (very early-onset IBD, VEO-IBD, defined as IBD onset before 6 years of age) underscores the urgent need for effective, well-tolerated therapies tailored to this population [2].

Currently, infliximab and adalimumab are the only biologic therapies specifically approved for pediatric UC, with well-documented use [3]. However, a subset of patients fail to achieve adequate disease control, even with off-label use of additional biologic treatments like vedolizumab and ustekinumab [4]. This therapeutic gap leaves ongoing inflammation unresolved, leading to significant morbidity and reduced quality of life [5].

To address this unmet need, interest has grown in exploring Janus kinase (JAK) inhibitors, which modulate immune responses differently from traditional biologics. JAK inhibitors represent a novel therapeutic class that targets intracellular signaling pathways involved in cytokine-mediated inflammation, specifically blocking the JAK-STAT pathway that is crucial for inflammatory cytokine signaling in IBD pathogenesis. Unlike biologics that target specific extracellular cytokines or their receptors, JAK inhibitors provide broader immunomodulation by simultaneously affecting multiple inflammatory pathways downstream of various cytokine receptors.

Upadacitinib, a selective JAK1 inhibitor with some JAK2, JAK3, and TYK2 activity, has demonstrated promising outcomes in adult populations at standard dosing of 45 mg once daily for induction and 15–30 mg once daily for maintenance [6]. The drug’s oral bioavailability and rapid onset of action make it an attractive therapeutic option, particularly for patients who have failed multiple biologic therapies.

However, upadacitinib currently lacks regulatory approval for pediatric IBD patients. In the absence of established pediatric dosing guidelines, clinicians typically extrapolate dosing from adult regimens based on body weight, body surface area, or pharmacokinetic modeling, though this approach remains empirical and requires careful monitoring. Emerging case reports and anecdotal evidence suggest upadacitinib may also be beneficial in children and adolescents with severe IBD, providing hope for those who have exhausted other treatments [1,2,7,8]. However, robust pediatric studies are still needed to establish its safety, efficacy, and optimal dosing in younger patients. Given these considerations, we initiated the use of upadacitinib in children and adolescents who had failed to respond to multiple biologic therapies in our centers. Upadacitinib was trialed in a cohort of 5 pediatric patients.

Notably, four patients in our cohort experienced rapid symptom relief and achieved clinical remission (defined as fecal calprotectin < 100 mg/kg and PUCAI < 10 or PCDAI < 10) demonstrating upadacitinib’s potential in pediatric IBD, even in biologic-refractory and younger patients. However, one patient (Patient 4) did not respond to the therapy, underscoring the variability in treatment outcomes and the need for individualized therapeutic strategies.

## 2. Cases

### 2.1. Patient 1

A 14-year-old female, diagnosed with UC at the age of 11 (PUCAI at diagnosis 65), presented with a long-standing history of treatment-refractory disease. Initial therapy, in accordance with established guidelines, included mesalazine, prednisolone, and azathioprine. Although a temporary clinical improvement was observed during corticosteroid treatment, symptoms recurred following prednisolone tapering, and sustained remission was never achieved [8,9].

**Figure 1:**
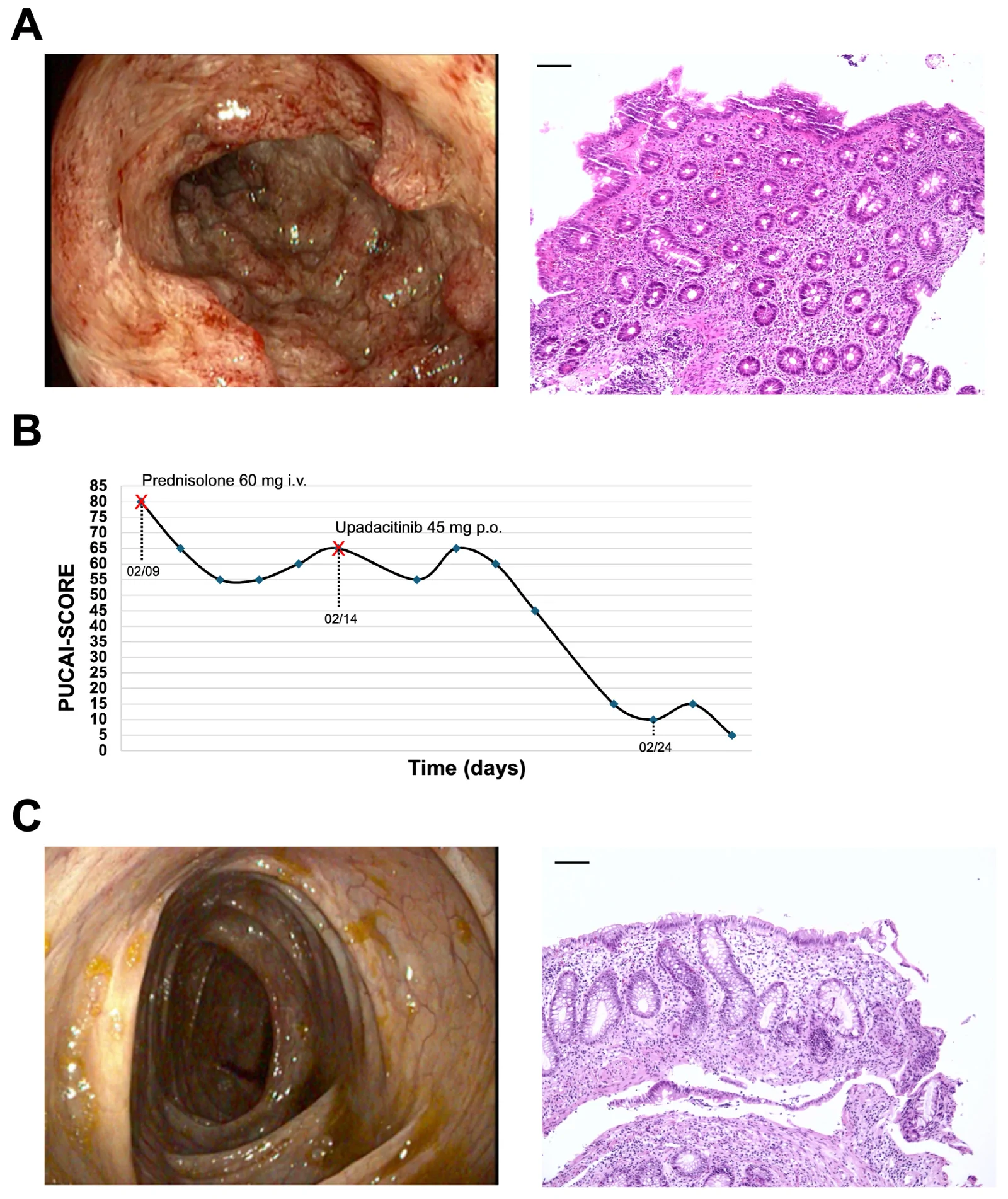
Disease Course of Patient 1 with Ulcerative Colitis. (**A**) Initial endoscopic (UCEIS 9) and histological findings of severe inflammation (erosive lesions, spontaneous bleeding, etc.) associated with ulcerative colitis during the first endoscopy. The histological image (H & E staining, 10×, Scalebar 100 µm) reveals a moderately dense infiltrate comprising lymphocytes, plasma cells, neutrophils, and eosinophilic granulocytes, along with mild crypt architectural distortion, indicative of moderate disease activity. (**B**) Temporal course of the Pediatric Ulcerative Colitis Activity Index (PUCAI) following hospitalization and commencement of upadacitinib therapy. (**C**) Endoscopic (UCEIS 0) and histological findings six months after initiating upadacitinib therapy demonstrate deep remission. The histological image (H & E staining, 10×, Scalebar 100 µm) shows minimal lymphocytic infiltrate with a few eosinophilic granulocytes, consistent with remission.

The patient failed to achieve sustained remission under multiple biologic therapies. infliximab was discontinued after a few months due to psoriasiform skin lesions and replaced by adalimumab, which also proved ineffective despite therapeutic drug levels. After six months, therapy was switched to vedolizumab, followed by ustekinumab—both without clinical benefit. During ustekinumab treatment, tacrolimus (3–6 mg/day) was intermittently added. A subsequent high-dose re-induction with infliximab again failed, prompting initiation of tofacitinib (10 mg orally once daily), which was also unsuccessful. Throughout this period, the patient suffered from transfusion-dependent anemia and required intermittent corticosteroid therapy. At an age of 15, she was then admitted with acute severe colitis, presenting with a Pediatric Ulcerative Colitis Activity Index (PUCAI) score of 80, anemia, C-reactive protein (CRP) 92.5 mg/L, calprotectin 1390 mg/kg, low-grade fever, and significantly reduced condition. Sonography revealed pancolitis, and endoscopy confirmed severe inflammation of the colonic mucosa (UCEIS 8, Mayo 3) (Figure 1A). At admission, she was on maintenance tofacitinib (10 mg orally twice daily) and tacrolimus (3 mg orally once daily).

Despite prior treatments, her condition remained critical. Following guidelines, she was transitioned to high-dose methylprednisolone (60 mg intravenously once daily), and colectomy was considered [8]. As a last effort, off-label rescue therapy with upadacitinib (45 mg orally once daily) was initiated, leading to rapid and significant improvement within days, avoiding surgery. Her PUCAI score dropped to 5–10 and has remained stable (Figure 1B). Calprotectin levels are consistently negative, and she is now in excellent health.

After 12 weeks, her upadacitinib dose was reduced to 30 mg daily, but mild symptoms recurred, prompting reinstatement of the original 45 mg dose. After 10 months of treatment, no significant side effects were observed, apart from occasional respiratory infections and mild herpes labialis. A 6-month of follow-up, endoscopy showed macroscopic mucosal healing, with normal histological findings, indicating deep remission (Figure 1C).

### 2.2. Patient 2

This case report details a 10-year-old boy diagnosed with severe UC at age of seven years and 10 months, whose disease has been highly resistant to standard treatments. Despite attempts to manage his condition with conventional therapies, responses were insufficient, necessitating multiple treatment escalations. Initially, he was prescribed mesalazine and azathioprine, but no clinical improvement was observed after 4 months. Persistent symptoms led to escalation to infliximab, a TNF-alpha inhibitor. While infliximab initially induced clinical remission, fecal calprotectin levels (250–400 mg/kg) indicated ongoing subclinical inflammation. Remission was short-lived, and symptoms worsened. At the age of 9, the patient was transferred to our hospital with a PUCAI score of 65, indicating moderate to severe disease activity. Colonoscopy revealed extensive, highly active colitis involving the entire colon (Paris E4, UCEIS 7). As a result, treatment was escalated to vedolizumab. However, after four months without clinical improvement (PUCAI still 45), therapy was switched to ustekinumab.

Ustekinumab initially showed promise, but long-term symptomatic relief was partial. The patient experienced 3–4 loose, bloody stools daily, with a PUCAI of 40–45, signaling inadequate disease control. After 4 months, treatment shifted to upadacitinib, a Janus kinase (JAK) inhibitor targeting an alternative inflammatory pathway.

Upadacitinib, initiated at 45 mg daily, resulted in sustained clinical remission maintained for 5 months. PUCAI dropped to 0 within the first month. However, reducing the dose to 30 mg daily caused a slight clinical deterioration, with PUCAI increasing to 15. Restoring the 45 mg dose led to clinical remission within days with PUCAI of 0.

### 2.3. Patient 3

This case report describes a 13-year-old boy diagnosed with CD at an age 10. The disease primarily affects his terminal ileum and colon, classified as A1b, L1, B1, G1 under the Paris classification with a PCDAI score of 7. At diagnosis, treatment began with infliximab. Despite high-dose therapy (10 mg/kg every four weeks), no clinical response was observed after six months, prompting a switch to adalimumab. However, after three months on adalimumab (40 mg every week) with adalimumab through levels of around 15 µg/mL, disease activity persisted, requiring further intervention.

Ten months post-diagnosis, endoscopy confirmed severe inflammation in the terminal ileum and colon (Paris A1b, L3, B1, G1; UCEIS 7–8). After failure of two infliximab and adalimumab, treatment shifted to ustekinumab yielding significant clinical improvement. Stool frequency decreased from 5–6 times daily to 1–2 times, and growth improved, suggesting better nutrient absorption. However, fecal calprotectin levels remained >1000 mg/kg after 12 months, indicating unresolved inflammation.

Follow-up endoscopy revealed a stricturing lesion at the ileocecal valve, indicating disease progression with structural complications. The patient started budesonide (9 mg daily), but after two months, inflammation persisted, and the lesion showed no improvement. Surgery became necessary, resulting in ileocecal resection. Post-surgery, while continuing ustekinumab, fecal calprotectin levels remained high, and subsequent endoscopy showed persistent ileal inflammation.

Given ongoing issues, treatment shifted to upadacitinib at 45 mg for four weeks. Within three weeks, fecal calprotectin levels fell below 250 mg/kg, and by six weeks, they dropped further to below 100 mg/kg, signaling reduced inflammation. However, reducing the dose to 30 mg caused symptom recurrence and an increase in fecal calprotectin to 340 mg/kg. Restoring the 45 mg dose regained disease control, with the patient remaining asymptomatic for nearly six months. Growth trajectory improved by +0.7 SDS, with fecal calprotectin levels ranging between 40 and 190 mg/kg. The current PCDAI score is 0. No follow-up endoscopy has been performed to date.

### 2.4. Patient 4

A now 17 1/2-year-old male was diagnosed with pancolitis ulcerosa at the age of 12 (PUCAI 60, UCEIS 6). Initial treatment consisted of mesalazine and azathioprine. However, this regimen failed to achieve adequate disease control. Three months post-diagnosis, infliximab was added. The patient developed an allergic reaction and anti-drug antibodies (ADAs) after the second infusion, necessitating the discontinuation of infliximab. adalimumab was then initiated in combination with mesalazine and azathioprine. Despite this adjustment, the patient demonstrated only a partial response, and ADAs against adalimumab were detected within the first year. As a result, adalimumab was discontinued. PUCAI score at this stage was 45.

Vedolizumab monotherapy was commenced, leading to sustained clinical remission for 2.5 years. This period of stability was interrupted in by a Campylobacter infection, followed by an infection with Clostridium difficile. These infections marked a turning point, as they destabilized the patient’s previously controlled colitis. Intensive glucocorticoid therapy (2 mg/kg/day prednisolone for one week followed by tapering over 8 weeks) and dose optimization of vedolizumab failed to restore disease control, prompting another change in therapy.

The then 17-year-old patient was started on upadacitinib at a dose of 45 mg once daily. While initial response was promising, with clinical remission achieved, the colitis flared again after three months. Despite continued high-dose upadacitinib therapy, disease activity persisted. Subsequently, Risankizumab was introduced. Risankizumab is a humanized monoclonal antibody that specifically targets the p19 subunit of interleukin-23 (IL-23), blocking the IL-23/IL-17 inflammatory pathway that plays a crucial role in the pathogenesis of inflammatory bowel disease. By inhibiting IL-23, risankizumab reduces the activation and proliferation of Th17 cells and the subsequent production of pro-inflammatory cytokines [10]. At the time of reporting, the patient’s colitis remains refractory, and permanent disease control has not yet been achieved.

### 2.5. Patient 5

An 8-year-old boy was diagnosed with pancolitis ulcerosa at the age of 3 years. Due to the very early onset of disease, next-generation sequencing (NGS) was performed to evaluate for monogenic inflammatory bowel disease. The panel included genes such as IL10, IL10RA, IL10RB, XIAP, FOXP3, LRBA, CYBB, CTLA4, TTC7A, among others (full gene list available upon request). No pathogenic variants were detected.

Initial treatment included mesalazine and azathioprine, followed by several courses of systemic corticosteroids over the first years after diagnosis. Due to persistent disease activity, biologic therapy with infliximab was initiated but discontinued after 6 months due to lack of response. Subsequently, adalimumab was administered for another 6 months without clinical improvement. This was followed by vedolizumab (6 months), multiple corticosteroid pulses, and a 7-month course of ustekinumab—all without achieving sustained remission.

Repeated endoscopic evaluations confirmed severe colonic inflammation (UCEIS 8, Mayo 3). To induce remission, high-dose tofacitinib (10 mg orally once daily) was introduced, resulting in only transient symptom relief.

Given the ongoing disease activity (PUCAI 55, fecal calprotectin 1070 mg/kg, normal CRP), treatment was escalated to upadacitinib at 30 mg once daily (body weight: 30 kg). Since initiation of upadacitinib, the patient has remained in sustained clinical remission, with complete resolution of gastrointestinal symptoms and normalization of fecal calprotectin. No adverse effects have been observed, and laboratory parameters have remained within normal limits throughout the treatment period.

## 3. Discussion

This case series provides valuable insights into the potential role of upadacitinib as an effective treatment for pediatric patients with severe, therapy-refractory IBD. Our findings align with the growing body of evidence suggesting that upadacitinib may serve as a viable therapeutic option in such cases, particularly for CD and UC [6,7,11,12,13,14]. Recent literature has reported promising outcomes in pediatric populations, further substantiating its clinical utility.

For instance, a recent case report described the successful use of upadacitinib in a child with an ATM (ataxia-teleangiectasia mutated protein) gene mutation and refractory CD, who achieved rapid remission with upadacitinib after multiple biologic therapies had failed [15].The ATM gene mutation is involved in DNA repair mechanisms and known to influence immune function and inflammatory pathways, therefore this case highlights a potential therapeutic advantage of JAK inhibitors like upadacitinib in genetically complex cases. Similarly, our 13-year-old patient with refractory CD demonstrated significant clinical improvement despite structural complications such as strictures, underscoring the drug’s efficacy even in anatomically challenging scenarios.

In UC, the effectiveness of upadacitinib has also been documented [7,11]. A report on VEO-IBD with refractory ulcerative colitis described rapid symptom control and mucosal healing [14], findings echoed in our VEO-IBD UC case where patient experienced substantial clinical improvements. These observations emphasize the potential of JAK inhibitors to address the therapeutic challenges posed by aggressive, early-onset disease phenotypes. Importantly, the report highlighted the importance of tailored dosing strategies for younger patients, a conclusion supported by our findings of dose-dependent disease control.

Further supporting evidence comes from a cohort study evaluating upadacitinib in refractory pediatric IBD [13]. This study demonstrated high rates of clinical and endoscopic remission in adolescents with acute severe UC, mirroring the outcomes observed in our patients. Additionally, the study reinforced the safety profile of upadacitinib, reporting only mild, manageable adverse effects such as herpes labialis—similar to the side effects seen in our cohort.

An emerging and particularly relevant aspect of upadacitinib therapy is its potential use in combination with biologic agents for refractory IBD. Recent evidence suggests that JAK inhibitors such as upadacitinib can be used as dual therapy, with favorable outcomes shown in nine patients using a combination of upadacitinib and ustekinumab or vedolizumab [13]. This dual targeted therapy approach may be particularly feasible in children with refractory disease, offering several potential advantages including synergistic mechanisms of action targeting different inflammatory pathways.

However, combination therapy also presents potential disadvantages that require careful consideration. The increased immunosuppressive burden may elevate risks of opportunistic infections and malignancies, particularly concerning in the pediatric population where long-term safety data remain limited. Additionally, drug–drug interactions, increased healthcare costs, and the complexity of monitoring multiple agents simultaneously present practical challenges. Our case series did not employ combination therapy, but future studies should systematically evaluate the risk-benefit profile of such approaches in pediatric IBD.

The potential of JAK inhibitors to serve as salvage therapy and prevent surgical interventions represents a critical advantage in severe IBD cases [7,16]. In our cohort, the 15-year-old UC patient who avoided colectomy after initiating upadacitinib exemplifies this therapeutic potential. Recent studies have specifically highlighted upadacitinib as an effective induction therapy for refractory pediatric UC, with efficacy that should be weighed against potential adverse events.

The ability to achieve rapid clinical improvement, even in post-surgical and stricture-associated cases as observed in our series, suggests that upadacitinib may alter the natural history of pediatric IBD by reducing the need for surgical interventions. However, the long-term impact on surgical rates requires prospective evaluation, as some patients may experience delayed complications despite initial therapeutic success.

The efficacy of upadacitinib in acute severe UC was also highlighted in another case series, which documented rapid reductions in PUCAI scores and mucosal healing [11]. These findings are consistent with the trajectories observed in our UC patients, including a 15-year-old girl who avoided colectomy after initiating upadacitinib. This underscores the potential of JAK inhibitors to serve as a salvage therapy, possibly preventing surgical interventions in severe cases.

In our cohort, no significant adverse events were observed across all patients during upadacitinib treatment. The only notable finding was occasionally increased frequency of herpes labialis in individual patients; however, these episodes represented exacerbations of pre-existing herpes simplex infections that were already present prior to upadacitinib initiation. This pattern is consistent with the known immunomodulatory effects of JAK inhibitors and aligns with the safety profile reported in recent pediatric literature, where herpes labialis has been identified as a manageable adverse effect [13]. The absence of severe complications in our series supports the favorable safety profile of upadacitinib in pediatric IBD patients, though long-term surveillance remains essential.

Our case series contributes further evidence to the growing recognition of upadacitinib as a promising treatment option for therapy-refractory pediatric IBD, particularly in early-onset and structurally complex cases. Notably, our patients were younger than those described in most previous studies, emphasizing upadacitinib’s potential efficacy in a younger patient cohort. The rapid and sustained clinical improvements observed, even in post-surgical and stricture-associated cases, highlight its versatility and therapeutic potential.

However, as a case series, this study has inherent limitations that must be acknowledged when interpreting these findings. The small sample size limits the generalizability of our results. Absence of a control group prevents direct comparison with alternative therapeutic approaches or natural disease progression. The relatively short follow-up duration, while sufficient to demonstrate short-term efficacy, limits our ability to assess long-term outcomes and safety profiles.

Furthermore, the need to maintain patients on higher-than-recommended doses to achieve disease control-given the consistent relapses observed with dose reductions-raises critical questions about optimal dosing strategies in pediatric populations that require validation in larger, prospective studies. Additionally, upadacitinib does not yield positive outcomes in all cases, as evidenced by our case series, further emphasizing the need for clear, evidence-based indicators to guide the selection of advanced therapies in pediatric IBD.

These limitations underscore the urgent need for larger, multicenter, randomized controlled trials to establish the efficacy, safety, and optimal dosing of upadacitinib in pediatric IBD populations and to provide the robust evidence base necessary for regulatory approval and clinical guideline development. Future studies should focus on long-term efficacy, safety, and tailored dosing strategies, while also developing predictive tools to optimize treatment selection for individual patients.

## Data Availability

The original contributions presented in this study are included in the article. Further inquiries can be directed to the corresponding author.

## Abbreviations

The following abbreviations are used in this manuscript:

ADA	Anti-drug antibodies
CD	Crohn’s disease
IBD	Inflammatory bowel disease
JAK	Janus kinase
PCDAI	Pediatric Crohn’s Disease Activity Index
PUCAI	Pediatric Ulcerative Colitis Activity Index
UC	Ulcerative colitis
UCEIS	Ulcerative Colitis Endoscopic Index of Severity
VEO-IBD	Very Early Onset Inflammatory Bowel Disease
